# Supplementary material for: Silicon Nano-Fertilizer-Enhanced Soybean Resilience and Yield Under Drought Stress
Source: Plants (Basel). 2025 Mar 1;14(5):751. doi: 10.3390/plants14050751 (PMC11902048; doi:10.3390/plants14050751)
Supplement: Supplementary file 1 [file plants-14-00751-s001.zip › plants-3417076-supplementary.pdf]

Supplementary file

Silicon Nano Fertilizer Enhanced the Soybean Resilience and Yield Under Drought Stress

Jian Wei[1] weijian@ccsfu.edu.cn, Liu Lu[2] liulujlnd@163.com, Wei Zihan[3] weizihan0011@outlook.com, Qin Qiushi[4] qiugiushi@csvw.com, Bai Qianyue[1] 14704377775@163.com, Zhao Chungang[1] zhaochungang1@outlook.com, Zhang Shuheng[1] 18686422696@163.com, Hongtao Wang[2*] hongtaowang@thnu.edu.cn

[1]School of Agriculture, Jilin Agricultural University, Changchun, 130118, China;

[2]College of Life Sciences, Tonghua Normal University, Tonghua, 950, Yucai Road, China;

[3]School of Life Sciences, Wuhan University;

[4]Jilin Changfa Modern Agricultural Technology Group Co., Ltd

[*]Correspondence: Hongtao Wang hongtaowang@thnu.edu.cn

Silicon based nano fertilizer enhanced the Soybean resilience and yield under drought stress

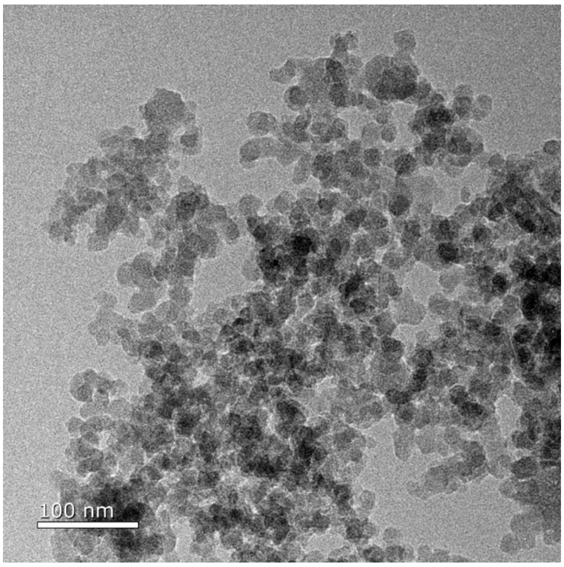

Figure S1. Transmission electron mission (TEM) image of nanoscale silicon oxide.

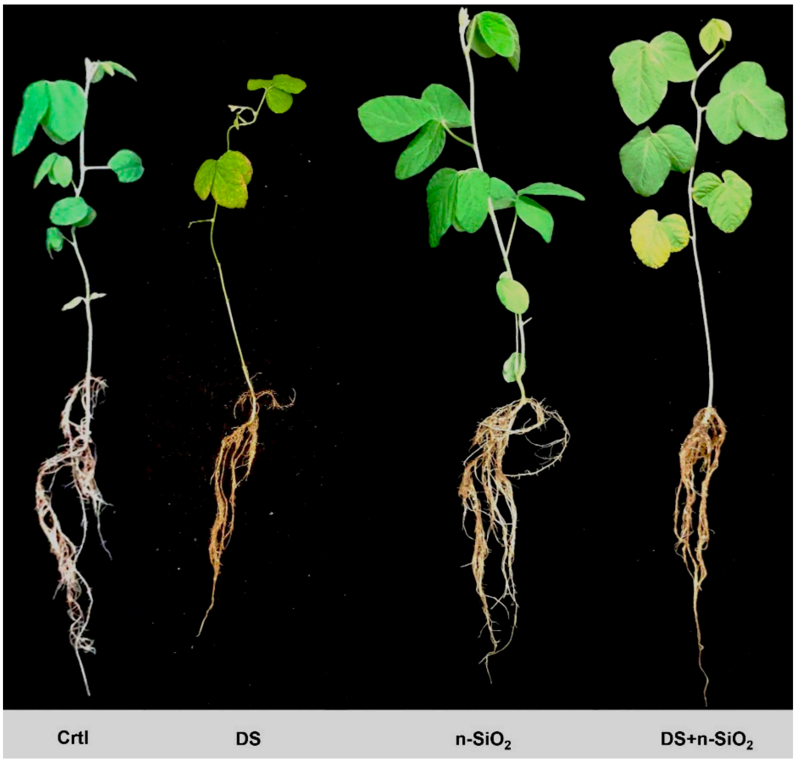

Figure S2. Effects of different treatments on soybean growth. Phenotypic images of soybean plants at 35 days old under four treatments at the vegetative stage of the first harvest: control (Ctrl), drought stress (DS), nano-silicon dioxide (n-SiO_2), and combined drought stress with nano-silicon dioxide (DS+n-SiO_2).

Table S1. The zeta-potential and hydrodynamic diameter of n-SiO_2. The data presented as the mean ± standard deviation (n=3)

	**n-SiO_2**
Zeta potential (MV)	-31 ± 1.6
Hydrodynamic diameter (nm)	243.5 ± 13.58

Table S2. Soil characteristics

Index	Mean value
Soil	
pH	7.4
Available K (mg kg^{-1})	154.8
Available phosphorus (mg kg^{-1})	38.5
Organic matter (mg kg^{-1})	25.3
Available Si (mg kg^{-1})	347.19
